# Doing No Harm? Adverse Events in a Nation-Wide Cohort of Patients with Multidrug-Resistant Tuberculosis in Nigeria

**DOI:** 10.1371/journal.pone.0120161

**Published:** 2015-03-17

**Authors:** Yohanna Kamabi Avong, Petros Isaakidis, Sven Gudmund Hinderaker, Rafael Van den Bergh, Engy Ali, Bolajoko Oladunni Obembe, Ernest Ekong, Clement Adebamowo, Nicaise Ndembi, James Okuma, Adeline Osakwe, Olanrewaju Oladimeji, Gabriel Akang, Joshua Olusegun Obasanya, Osman Eltayeb, Aderonke Vivian Agbaje, Alash’le Abimiku, Charles Olalekan Mensah, Patrick Sunday Dakum

**Affiliations:** 1 Institute of Human Virology, Abuja, Nigeria; 2 Médecins Sans Frontières, Operational Research Unit, Luxembourg City, Luxembourg; 3 Centre for International Health, University of Bergen, Bergen, Norway; 4 University of Maryland School of Medicine, Baltimore, Maryland, United States of America; 5 National Agency for Food and Drug Administration Control, Abuja, Nigeria; 6 Zankli Medical Centre, Abuja, Nigeria; 7 Liverpool School of Tropical Medicines, Pembroke Place, Liverpool, United Kingdom; 8 National Tuberculosis and Leprosy Control Program, Abuja, Nigeria; 9 Damien Foundation Belgium, Nigeria Project, Ibadan, Nigeria; College of Medicine, University of Ibadan, University College Hospital, Ibadan, Nigeria., NIGERIA

## Abstract

**Background:**

Adverse events (AEs) of second line anti-tuberculosis drugs (SLDs) are relatively well documented. However, the actual burden has rarely been described in detail in programmatic settings. We investigated the occurrence of these events in the national cohort of multidrug-resistant tuberculosis (MDR-TB) patients in Nigeria.

**Method:**

This was a retrospective, observational cohort study, using pharmacovigilance data systematically collected at all MDR-TB treatment centers in Nigeria. Characteristics of AEs during the intensive phase treatment were documented, and risk factors for development of AEs were assessed.

**Results:**

Four hundred and sixty patients were included in the analysis: 62% were male; median age was 33 years [Interquartile Range (IQR):28–42] and median weight was 51 kg (IQR: 45–59). Two hundred and three (44%) patients experienced AEs; four died of conditions associated with SLD AEs. Gastro-intestinal (n = 100), neurological (n = 75), ototoxic (n = 72) and psychiatric (n = 60) AEs were the most commonly reported, whereas ototoxic and psychiatric AEs were the most debilitating. Majority of AEs developed after 1–2 months of therapy, and resolved in less than a month after treatment. Some treatment centers were twice as likely to report AEs compared with others, highlighting significant inconsistencies in reporting at different treatment centers. Patients with a higher body weight had an increased risk of experiencing AEs. No differences were observed in risk of AEs between HIV-infected and uninfected patients. Similarly, age was not significantly associated with AEs.

**Conclusion:**

Patients in the Nigerian MDR-TB cohort experienced a wide range of AEs, some of which were disabling and fatal. Early identification and prompt management as well as standardized reporting of AEs at all levels of healthcare, including the community is urgently needed. Safer regimens for drug-resistant TB with the shortest duration are advocated.

## Introduction

Multidrug-resistant tuberculosis (MDR-TB) (Tuberculosis that is resistant to at least Rifampicin and Isoniazid) is a growing clinical and public health problem worldwide [1, 2]. Treatment takes a total duration of 20 months (8 months intensive phase inclusive) of administration of second line anti-tuberculosis drugs (SLDs), which are associated with a wide range of adverse drug reactions (ADRs) [3,4]. In recent times, such ADRs have become a source of global public health concern, since they significantly contribute to morbidity, mortality, losses to follow-up and increased health care costs [3–7]. In resource-limited settings, the consequences of ADRs is alarming because the health facilities and specialist services required for the management are scarce; hence, treatment interruptions, adherence difficulties, loss to follow up, treatment failure and as a consequence, the emergence of further drug resistance [6–9] are expected. For this reason, the World Health Organization (WHO) has recommended the aggressive management of ADRs as an essential component of the care of MDR-TB patients [10].

Nigeria’s total population in 2014 was above 170 million people spread across 36 states and the Federal Capital Territory (FCT) [11]. Approximately 50% of the total population lives in urban areas. In 2014, the population growth rate and life expectancy at birth for the total population were 2.47% and 52.62 years respectively. Hospital bed density in 2004 was 0.53 beds per 1000 population, while maternal mortality rate was 630 deaths per 100,000 live births in 2014 [11]. Infant mortality rate was 74.04 deaths per 1,000 live births in 2014[11]. In the midst of HIV/AIDS epidemics, MDR-TB has emerged as a significant public health problem. The 2012 National Drug Resistant Tuberculosis Prevalence Survey (NDRPS) revealed a growing population of people living with MDR-TB. At least, 2.9% of reported MDR-TB cases were from new TB patients while 14.3% were from previously treated TB patients [2, 12]. However, according to government sources, notable achievements have been recorded in controlling the epidemic. Some of these achievements include the development of the Nigerian MDR-TB National Treatment Guidelines [13], the award of the Global Fund Round 9 grant (GFR9) for the control of MDRTB and case management of MDR-TB patients at health facilities up to the Local Government Areas (LGAs) level.

Drug-susceptible TB patients, including those with a previous history of TB treatment, initiate treatment with first-line anti-TB drugs at one of the 3,455 health facilities in Nigeria [14]. Once these patients are diagnosed with MDR-TB, they are registered and referred to the MDR-TB treatment centers by the national tuberculosis and leprosy control program (NTBLCP) after being educated on the need for long hospitalization and have signed informed consent forms. There were nine functional MDR-TB centers spread across seven states as of 31^st^ March 2014. The number of treatment centers is however expected to multiply in the next few years because the GeneXpert MTB/RIF technology, which facilitates early detection of MDR-TB among patients in high risk group, has been introduced. In 2013, there are over 64 GeneXpert POC Machines for molecular diagnosis, distributed all over the states.

Despite the remarkable achievements that have been recorded, there is no published report on the AEs associated with the SLDs in Nigeria. Local studies have focused on issues related to the under-reporting of the AEs to the National Pharmacovigilance Center (NPC) [15–18] without assessing the actual burden of AEs, and as a result, there is a paucity of AEs data to promote patient care as well as national drug safety surveillance. At the global level, the few studies that have reported the AEs of the SLDs included low numbers of participants from individual treatment centers, which limited the generalizability of the studies [19–21]. Our study has addressed these gaps in knowledge in two main ways: we relied on the national cohort of MDR-TB patients from the nine treatment centers, and assessed the actual burden of AEs during the intensive phase of treatment over a period of two years. The objectives of the study were to document the incidence, types, time of occurrence, and duration of AEs among MDR-TB patients during the intensive phase of treatment and their risk factors.

## Methods

### Definition of terms

In describing the consequences of the SLDs, we utilized two terms: “adverse drug reactions” and “adverse event”. Adverse drug reaction (ADR) according to the WHO, is any noxious and unintended response to a drug that occurs at doses normally used in man to prevent, diagnose, or treat disease or to modify physiological function [5], while “adverse event” (AE) refers to: (1) Any untoward medical occurrence in a patient or clinical investigation subject given a pharmaceutical product; does not necessarily have a causal relationship with such treatment; and (2) Any unfavorable and unintended sign (including abnormal laboratory findings), symptom, or disease temporally associated with the use of a medicinal (investigational) product; not necessarily related to the product [22].

### Ethics

Ethical approval was given by the Health Research Ethics Committee of the Institute of Human Virology Nigeria in collaboration with the National Health Research Ethics Committee of Nigeria in August, 2013. This study has also met the Médecins Sans Frontières Ethics Review Board (Geneva, Switzerland) approved criteria for analysis of routinely-collected program data in August, 2013. It satisfies the requirements of the Ethics Advisory Group of the International Union Against Tuberculosis and Lung Disease, Paris, France. Patient information was anonymized and de-identified prior to analysis. As this was a routinely collected program data, informed consent from the patients was not obtained. The named ethics committees approved the study and waived the need for consent.

### Study design

This was a retrospective, observational cohort study, using pharmacovigilance data systematically collected at all MDR-TB treatment centers in Nigeria.

### Study population

All MDR-TB patients starting the intensive phase treatment at all MDR-TB treatment centers in Nigeria, from 1^st^ February 2012 to 31^st^ December 2013, were included in the study.

### Setting

**MDR-TB Program in Nigeria**. Treatment of MDR-TB consists of an intensive and a continuation phase under direct observation of trained health care providers. The intensive phase is strictly hospital-based during which patients are admitted and given a standard regimen consisting of Kanamycin (Km) or Amikacin (Amk), Pyrazinamide (Z), Levofloxacin (Lfx), Cycloserine (Cs), Prothionamide (Pto) and Pyridoxine, for at least eight months. This is followed by twelve months of continuation phase during which patients take Pyrazinamide (Z), Levofloxacin (Lfx), Cycloserine (Cs), Prothionamide (Pto) and Pyridoxine.

Clinical and bacteriological monitoring using sputum smear/culture tests is performed monthly during the intensive phase and bimonthly during the continuation phase. A patient is considered sputum smear or culture negative when results of two consecutive results are negative. Patients are discharged to the community where they are followed up on a regular basis at the end of the intensive phase. However, patients who remain culture positive at the end of the intensive phase, continue with the intensive phase treatment.

### Diagnosis of adverse events

AEs were diagnosed by the treatment centers through a combination of direct observation, laboratory report and participant’s reports—these methods are recommended by the international conference on harmonization (ICH) [22–24]. In the direct observation, a trained health worker (usually a clinician) physically examined the patient, while the laboratory reports involved conducting specific laboratory tests to diagnose or confirm a suspected AE. Participants’ reports involved the patients reporting the AEs they experienced to the health workers while on admission. Because of the subjective nature of the participants’ reports, clinicians requested for further investigations but the decision was entirely at the discretion of the clinician.

### Grouping and severity of adverse events

The AEs were grouped into 11 major classes based on recommendations from experts and ICH guidelines (Table 1).

**Table 1:** Grouping of adverse events of second—line anti-tuberculosis drugs.

CATEGORY	ADVERSE EVENTS
**OTOTOXICITY**	Tinnitus, Hearing loss, Deafness, Disequilibrium, Vertigo
**PSYCHIATRIC**	Irritability, Anxiety, Depression, Suicidal Ideation, Personality Changes, Depression, Psychosis,
**NEUROLOGICAL**	Dizziness, Insomnia, Vertigo, Convulsion, Syncope, Peripheral neuropathy, Blurring of Vision, Parasthesia, Fasciculation, Numbness, Incoherent speech, Oculogyric syndrome, Seizures, Palpitation, Atrophy at injection site
**ENDOCRINE**	Poor glycemic control, Hypothyroidism
**DERMATOLOGICAL**	Skin pigmentation, Photosensitivity, Dry skin, Stevens-Johnson syndrome, Fungal Infection, Itching, Skin reaction, Urticaria
**GASTROINTESTINAL**	Nausea, Vomiting, Gastritis, Ulcers, Hepatitis, Bowel obstruction, Abdominal discomfort, Gastrointestinal bleeding, Abdominal pain, Abdominal upset, Anorexia, Diarrhea, Dyspepsia, Hepatotoxicity
**ELECTROLYTE ABNORMALTIES**	Dehydration, Hypocalcaemia, Hypomagnesaemia, Hypokalaemia
**NEPHROLOGICAL**	Renal Insufficiency
**ALLERGIC**	Allergic reactions
**ARTHRALGIA**	Joint pain, Ankle swelling and pains, Arthritis, Myalgia, Stiffness of fingers
**GENERAL BODY PAIN**	Pain in the lower back, headache, pain at injection site, Generalized body pain, musculoskeletal pain, Pain radiating to the back.
**OTHERS**	Weight loss, Vaginal discharges, Anemia, Body weakness, Palpitation, Fever, Hair loss, Bitterness of mouth, Heart failure, Hypertension, No menstruation, Pains in the legs, Restlessness, Tendinitis, Unstable temperature.

The grouping of the AEs became necessary because patients took more than one drug and each drug presented with several AEs. We also categorized the severity of the AEs into 6 categories based on signs and symptoms (Table 2).

**Table 2:** Categorizing of adverse events based on signs and symptoms.

**None**	No signs/symptoms or within normal limits
**Mild**	Minor signs/symptoms; no specific medical intervention required; asymptomatic laboratory findings only, radiographic findings only; marginal clinical relevance
**Moderate**	Requiring minimal, local, or noninvasive intervention only
**Severe**	Significant symptoms requiring hospitalization or invasive intervention
**Life-threatening or disabling**	Complicated by acute, life-threatening metabolic or cardiovascular complications (such as circulatory failure, hemorrhage, sepsis); life-threatening physiological consequences; or need for intensive care or emergent invasive procedure
**Fatal**	Causing death

### Reporting of AEs at the treatment centers

**Fig 1:**
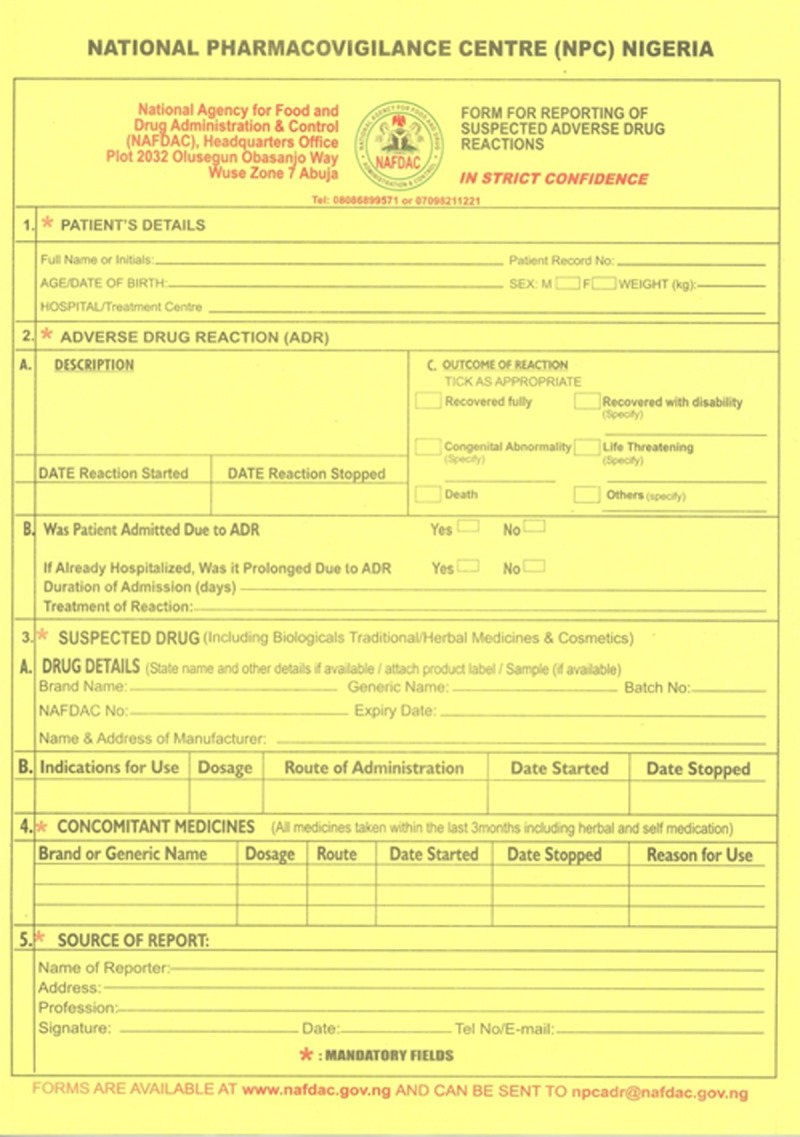
Pharmacovigilance Form (Yellow Form).

Suspected AEs were reported in the “Yellow Form” (Fig. 1) [also known as the Pharmacovigilance Form (PVG)] through the spontaneous reporting systems (SRS) [25–27]. The SRS is managed nationally by the National Pharmacovigilance Center located in the head office of the National Agency for Food and Drug Administration Control (NAFDAC) in Abuja.

The completed forms were collected and signed by the pharmacists and then dispatched as individual case safety reports (ICSRs) [28] to the NTBLCP, NPC and the Institute of Human Virology, Nigeria. In this study, we focused on AEs reported during the intensive phase since the patients were on admission at this time and were under observation by the health workers.

### Data collection and analysis

We collected the following variables from the completed PVG forms: unique patient identification number, age, gender, body weight, treatment centers, patient HIV status and AEs. We also collected the date of start of treatment; date of onset and duration of AEs for patients whose AEs had been treated and resolved at the time of data collection. The outcomes at the end of the intensive phase (completion of the intensive phase treatment, absconding from treatment and death) were also collected. We applied summary statistics to describe socio-demographic and treatment characteristics, while associations between the occurrence of AE and demographic and clinical characteristics were explored through multivariate logistic regression using a stepwise backward elimination approach; p-values based on Wald’s test are indicated.

## Results

Of the 473 MDR-TB patients who were registered for the intensive phase treatment during the review period, 13 (3%) were wrongly diagnosed as having MDR-TB, and were excluded from further analysis. Among the 460 included patients, 203 (44%) experienced AEs.

### Patient and adverse events characteristics

The socio-demographic and treatment characteristics are presented in Table 3. Male participants represented 62%; median age and weight were 33 years and 51 kg respectively. Ten percent of the patients were co-infected with the Human Immunodeficiency Virus (HIV).

**Table 3:** Baseline characteristics of MDR-TB patients admitted to in-patient care, Nigeria, 2012–2013 (N = 482).

Variable	Number (%)
**TOTAL**	460
**Treatment center**	
Dr. Lawrence Henshaw Memorial Hospital, Calabar	51 (11)
General Chest Hospital, Ibadan	82 (18)
Infectious Disease Hospital, Kano	40 (9)
Jos University Teaching Hospital, Jos	29 (6)
Mainland Hospital, Yaba	97 (21)
National Tuberculosis and Leprosy Training Center	41 (9)
University College Hospital, Ibadan	69 (15)
University of Port Harcourt Teaching Hospital	38 (8)
University of Uyo Teaching Hospital, Uyo	13 (3)
**Age (Years)**	
<15	6 (1)
15–25	79 (17)
26–35	187 (41)
36–45	98 (21)
46–55	60 (13)
56–65	23 (5)
>65	5 (1)
Not recorded	2 (0.4)
Median (IQR)	33 (28–42)
**Gender**	
Male participants	285 (62)
Female participants	172 (37)
Unknown	3 (1)
**Weight (Kg)**	
<25	6 (1)
25–30	4 (1)
31–60	355 (77)
61–90	94 (20)
Unknown	1 (0.2)
Median (IQR)	51 (45–59)
**HIV status**	
Negative	413 (90)
Positive	47 (10)

**Fig 2:**
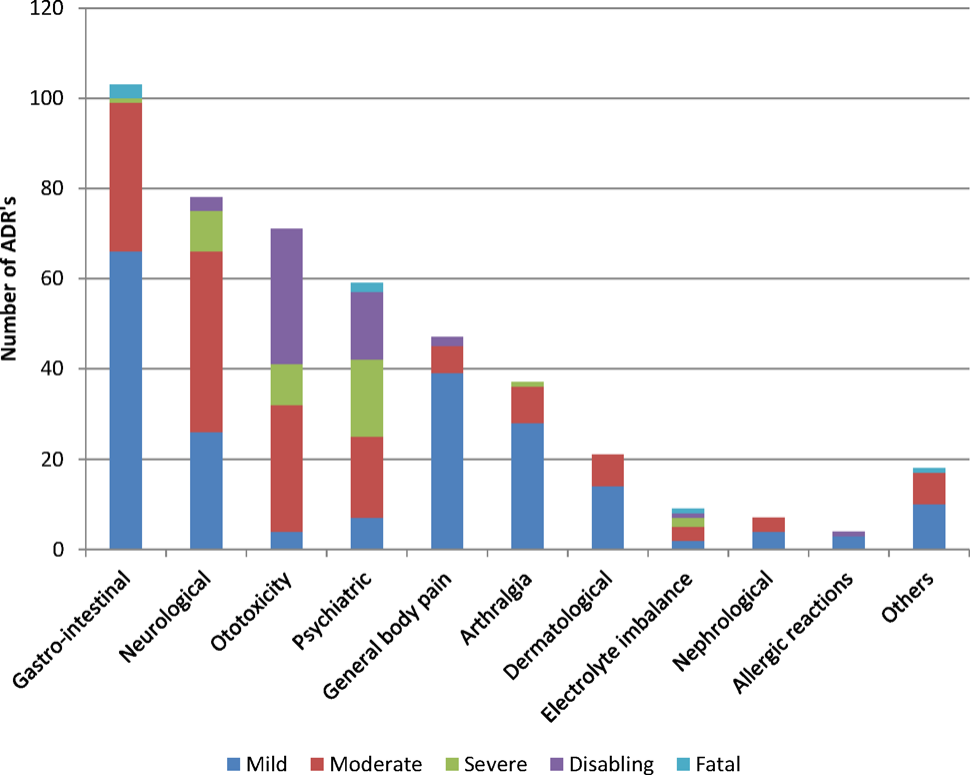
Number and severity of adverse events among MDR-TB patients in Nigeria, 2012–2013 (N = 482).

Fig. 2 shows the occurrence of the AEs and their severities; four patients died as a consequence of conditions associated with AEs (gastrointestinal = 2 deaths; electrolyte imbalance = 1 death and others (heart failure) = 1 death). Gastro-intestinal AEs were most commonly reported, followed by neurological, ototoxic and psychiatric AEs.

**Fig 3:**
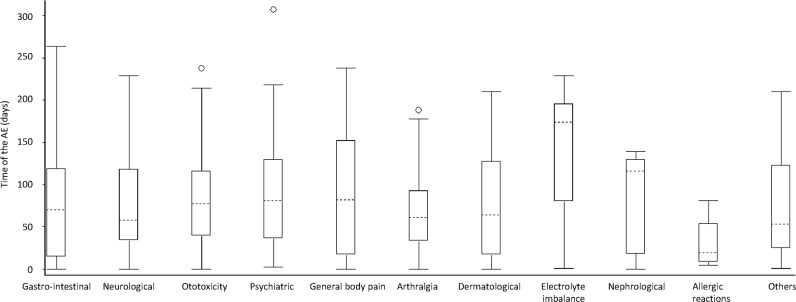
ADRs and time of onset among MDR-TB patients admitted to in-patient care, Nigeria, 2012–2013.

The most debilitating AEs were ototoxic and psychiatric events. In terms of timing of AEs occurrence, the first AE to appear were allergic reactions (at a median time of 20 days, IQR 5–81), while adverse events related to electrolyte imbalances took a longer time to appear (median 174 days, IQR 105–201) (Fig. 3).

**Fig 4:**
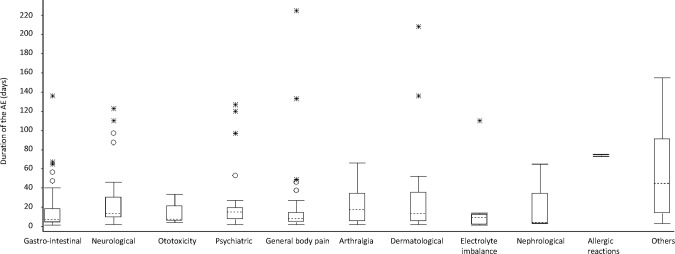
Duration of ADRs among MDR-TB patients admitted to in-patient care, Nigeria, 2012–2013.

In terms of duration, most AEs resolved in less than three weeks after onset (median 13 days, IQR 5–29) (Fig. 4).

At the end of the eight month intensive phase treatment, among the 373 patients with a follow up >8 months, 327 (88%) patients were alive; 21 (6%) died with the MDRTB while on treatment; 4 (1%) absconded without being discharged; 2 (1%) were discharged against medical advice and 2 (1%) developed extensively drug resistant tuberculosis (XDR-TB).

### Factors associated with adverse events

Potential associations between AEs and socio-demographic and/or clinical factors were analyzed: bivariate and multivariate analysis was performed. The same variables were identified as significant in bivariate and multivariate analysis; only the results of the multivariate analysis are shown (Table 4). Compared to the treatment center at the Maryland Hospital Yaba (MHY), the Dr Lawrence Memorial Hospital (DLMH) and University of Port Harcourt Teaching Hospital (UPTH) centers were more likely to report AEs [respectively OR 5.8 (95% CI 2.6–12.9; p<0.001) and OR = 8.6 (95%CI 3.0–24.1; p<0.001)]. Patients with a body weight of 61 kg to 90 kg had higher risk of AEs compared to patients with a body weight of 30 kg to 60 kg [OR 2.0 (95% CI 1.2–3.4; p = 0.008]. No increased risk of AEs was observed among HIV infected patients and no statistical associations were found between the AEs and intensive phase outcomes. We also did not observe any association between the AEs and age.

**Table 4:** Association between AEs, sex, age and body weight among MDR-TB patients admitted to in-patient care, Nigeria, 2012–2013.

Variable	AE	No AE	Adjusted OR (95% CI)*	Adjusted p value*
**TOTAL**	203 (44)	257 (56)	-	-
**Treatment center**				
MHY	40 (41)	57 (59)	1	-
DLHMH	41 (80)	10 (20)	5.8 (2.6–12.9)	<0.001
GCH	40 (49)	42 (51)	1.4 (0.8–2.5)	0.3
IDH	7 (18)	33 (83)	0.3 (0.1–0.8)	0.02
JUTH	7 (24)	22 (76)	0.4 (0.2–1.1)	0.09
NTBLTC	3 (7)	38 (93)	0.1 (0.04–0.4)	0.001
UCH	31 (45)	38 (55)	1.1 (0.6–2.0)	0.8
UPTH	33 (87)	5 (13)	8.6 (3.0–24.1)	<0.001
UUTH	1 (8)	12 (92)	0.1 (0.01–0.9)	0.04
**Sex**				
Male	118 (41)	167 (59)	1	-
Female	83 (48)	89 (52)	1.2 (0.7–1.9)	0.5
Not registered	2 (67)	1 (33)	-	-
**Age (Years)**				
<15	1 (17)	5 (83)	0.2 (0.01–3.5)	0.2
15–25	34 (43)	45 (57)	1.2 (0.6–2.2)	0.6
26–35	79 (42)	108 (58)	1	-
36–45	48 (49)	50 (51)	1.4 (0.8–2.4)	0.3
46–55	24 (40)	36 (60)	0.6 (0.3–1.2)	0.1
56–65	14 (61)	9 (39)	1.8 (0.7–4.8)	0.3
>65	2 (40)	3 (60)	0.9 (0.1–9.1)	0.9
Not recorded	1 (50)	1 (50)	-	-
**Weight (Kg)**				
<2525–30	2 (33)4 (100)	4 (67)0	1.0 (0.2–5.9)-	0.99-
31–60	141 (40)	214 (60)	1	-
61–90	56 (60)	38 (40)	2.0 (1.2–3.4)	0.008
Not recorded	0	1 (100)	-	-
**HIV status**				
Negative	182 (44)	231 (56)	1	-
Positive	21 (45)	26 (55)	1.7 (0.8–3.3)	0.2

* *Adjusted Odds Ratio and p-value (Wald’s test) based on multivariate logistic regression using a stepwise backward elimination approach*.

*Note*: *Treatment centers*: *DL HMH = Dr*. *Lawrence Henshaw Memorial Hospital*, *Calabar; GCH = General Chest Hospital*, *Ibadan; IDH = Infectious Disease Hospital*, *Kano; JUTH = Jos University Teaching Hospital*, *Jos; MHY = Mainland Hospital*, *Yaba; NTBLCT = National Tuberculosis and Leprosy Training Center; UCH = University College Hospital*, *Ibadan; UPTH = University of Port Harcourt Teaching Hospital; UUTH = University of Uyo Teaching Hospital*, *Uyo*.

## Discussion

This is the first study to report the burden of AEs of SLDs in Nigeria, even though the nation has been treating patients using these medications since June 2010 [14]. Previously published studies on MDR-TB focused either on the prevalence of MDR-TB or MDR-TB treatment outcomes, without evaluating the occurrence of AEs in great detail [29, 30]. We show a considerable burden of AEs in the MDR-TB population, with almost half suffering from AEs and many experiencing severe, debilitating and even fatal events.

Our study has four important findings. Firstly, we reported a wide range of AEs among the participants, which confirmed existing knowledge on the toxicity of SLDs reported in several studies ([3, 16, 17] and [31]). This finding echoes the overriding importance of managing AEs concurrently with MDR-TB treatment, which the WHO has stressed [8]. Secondly, we have identified the frequently experienced AEs in the Nigerian cohort (gastrointestinal, ototoxic, psychiatric and neurological); health workers should be alert for these AEs. Thirdly, we found significant variations in the reporting of AEs among the treatment centers, suggesting the need for standardization in training for all health staff. Fourthly, co-infection with HIV did not significantly increase the risk of AEs, which is consistent with findings from other studies ([19, 20] and [32]). We also found a high rate of patient survival and retention in care, which Oladimeji et al. [30] also reported.

The study had several strengths: it was country wide, and findings are thus likely to be generalizable. Additionally, we adhered to the STROBE guidelines [33]. However, we also faced some limitations: health workers may have wrongly diagnosed some AEs, which potentially affected the magnitude of the AEs in the studied population. Our choice of restricting the investigation to only the intensive phase treatment did not allow the investigation of the chronic AEs that might have appeared during the continuation phase treatment. However, despite these limitations, our findings have generated information with implications for policy and practice, mainly at the level of management of the AEs in hospital settings and ambulatory care, the need for safer drugs, management of co-morbidity and under-reporting of AEs to the NPC.

### Management of AEs in hospital settings

Our study and others [19, 20] suggest that gastro-intestinal, neurological, ototoxic and psychiatric AEs are likely to be experienced by many patients as they embark on the intensive phase treatment using the standard regimen. To minimize the adverse impact of AEs on treatment adherence, it is important that health staff are adequately trained on their recognition and management. Such training should include how to provide concise pretreatment counseling to patients on possible AEs of treatment [34]. It is also important that medications for managing AEs should be ordered concurrently with the ordering of SLDs to facilitate timely and adequate treatment of such AEs. However, limitation of funds can hinder the stocking of all ancillary drugs in addition to the SLDs: our findings can help guide which drugs to procure as a matter of priority.

### Management of AEs in ambulatory care

The NTBLCP currently manages the intensive phase of all MDR-TB patients in an inpatient model [30]. In not a distant future, Nigeria may transit to the ambulatory care model where patients are treated in the community in an effort to scale up patients’ recruitment. The delays caused by the limited inpatient capacity potentially increase the rate of TB mortality in the country. However, the magnitude and severity of AEs reported in this study suggest that the Federal Government needs to match the proposed transition with the scaling up of care for patient safety. Some of the severe, debilitating and fatal AEs reported in this study are better managed in specialist hospitals where qualified health professionals and services are readily available. An effective referral system for transferring patients from the communities to the hospitals needs to be in place once the community model of care commences. It is only then patients suffering from the AEs will have access to qualitative care.

### The need for safer drugs with shorter duration of treatment

The wide range of AEs reported in this study has elaborated and refined the well-known and dramatic toxicity of SLDs frequently reported in studies ([3, 4, 19] and [20]). Our findings support the call for safer and modified regimens, which the global plan to “Stop TB, 2011–2015” has emphasized [35]. Fortunately, there is an emerging body of studies highlighting the safety of some newer drugs or different combinations of existing drugs over the current SLDs. Examples are the new drug delamanid—a nitro-dihydro-imidazooxazole class of compounds—or the combination, nitroimidazo-oxazine, (coded as PA-824), moxifloxacin and pyrazinamide [36,37].

Some of the newer drugs have not been approved by the WHO but the real limitation is their cost. One tablet of delamanid 25mg was sold at US$295.00 in the open market as of 12^th^ June, 2014 [38], while a packet of 100 tablets of Levofloxacin 500mg sold between US$ 12:00 and US$16:00 through the Global Drug Facility (GDF) [39]. Since the newer drugs are still under patent, which may not allow cheaper generic brands to be manufactured, the Federal Government may need to sign commercial agreements with the manufacturers for the production of the generic brands. Additionally, the GFATM and other funding agencies need to increase funding for the MDR-TB program in Nigeria to enable the procurement of the newer drugs when finally approved.

### Management of co-morbidity

Drug interactions and AEs have been a major concern in treating MDR-TB patients who are concurrently infected with the Human Immunodeficiency Virus (HIV). One study reported that the side-effects of TB chemotherapy is magnified in patients with concurrent HIV treatment [40]. However, our study and others’ ([19, 20] and [32]) did not observe significant difference in the risk of AEs between HIV infected and non-HIV infected MDR-TB patients. Thus, the concern for AEs should not discourage the treatment of patients with the HIV/MDR-TB co-morbidity, although the drug-drug interactions still have to be considered.

### Underreporting of adverse events to the National Pharmacovigilance Center

Local drug safety surveillance for the protection of human populations from the dangers of AEs depends on an efficient reporting of ICSRs to central coordinating bodies like the NPC [41]. However, our evidence and that of other studies [15–18, 28] suggest a significant under-reporting of AEs to the NPC, as evidenced by the major differences in AE reporting between the treatment centers. We recommend the standardization of training so that all treatment centers can monitor, treat and report AEs to the NPC. Some local studies argued that lack of capacity is the primary factor inhibiting the reporting of the AEs [16–18]. Osakwe et al [28] found correlation between the training of health staff and reporting of AEs by health workers. Nigeria also needs to adopt the measures used in some countries to improve the reporting of AEs to the NPC. In Sweden, France and Italy, reporting of AEs is compulsory [42]. In the USA and more recently in some other countries, patients can also report directly to the spontaneous reporting system (SRS) [43].

### The need to increase patient enrollment into care

Almost 90% of the patients completed the intensive phase treatment and were alive at the time the data were collected; 21 (6%) patients died of TB while on treatment and 4 (1%) absconded without being discharged. In a similar study, Oladimeji et al reported a high rate of MDR-TB patient survival, “no loss to follow up” and the death of only 24 patients while on the intensive phase treatment [30]. The MDR-TB program may be doing well in Nigeria, but, there is need to match the high quality care with high patient enrollment, which appears to be far less than the WHO annual target of enrolling 3600 patients.

In conclusion, the participants experienced a wide range of AEs with a number of fatalities, thereby supporting existing evidence that SLDs are toxic drugs, demanding close monitoring of the patients. Further research is needed to assess the associations between the AEs and treatment outcomes.

We recommend early identification and prompt management of AEs. A standardized reporting of all AEs at all levels of healthcare, including the community should become a priority. We advocate for safer regimens for drug-resistant TB with the shortest duration possible. These are the minimum and immediate actions the government must take to protect the citizens from the harmful consequences of AEs.
